# Editorial Note: Metabarcoding analysis of eukaryotic microbiota in the gut of HIV-infected patients

**DOI:** 10.1371/journal.pone.0324348

**Published:** 2025-05-09

**Authors:** The PLOS One Editors

This Editorial Note provides an update to the previous Expression of Concern published on the linked article [1,2].

Following the publication of the article and Expression of Concern [1,2], PLOS investigated concerns pertaining to the reported ethical approval and the article’s adherence to *PLOS One*’s research ethics and reporting requirements.

Specifically, the research ethics concerns included that the study involved human participants but did not receive Comité de Protection des Personnes ethics approval, and the ethics approval number #2016–011 cited in [1] was also reported in > 50 other published articles despite apparent differences in the aims and objectives, study locations, study populations, age ranges, methodologies, types of samples collected, and types of consent described in these studies. S1 File contains a summary of articles citing ethics approval number #2016–011 of which PLOS is aware.

In addition, the *PLOS One* article [1] did not report sufficient information about participant recruitment and eligibility criteria as would be needed to replicate this study, and it did not report when the samples used in this study were collected.

A representative of the Aix-Marseille Université Ethics Committee stated that the institutional investigation into the ethics concerns concluded this article meets ethical standards. They commented that the stool samples collected in this study are considered human waste, and that the study did not require ethics approval from a Comité de Protection des Personnes according to French law. Furthermore, the representative indicated that they were not concerned about the ethics approval number reuse and stated that #2016–011 is a “generic” approval for a long-term global research project involving a series of retrospective laboratory tests on biological samples collected during routine patient care.

The ethics approval (#2016–011) was issued by the Ethics Committee of the IHU Mediterranean Infection and Institut Fédératif de Recherche 48 on September 21, 2016. The approval is for an epidemiological study of human microbiota using culturomics and metagenomics. It approves use of anonymized stool samples but does not mention HIV-infected patients or include other study-specific details such as approved study dates, sample sizes, or a description of the participant population(s).

Overall, the information and documentation received from Aix-Marseille Université support that the study abided by local ethics requirements. However, some issues remain unresolved:

– In response to editorial queries the authors did not clarify when the samples used in this study [1] were collected. This information is needed to evaluate the article’s compliance with the PLOS Human Subjects Research policy.
– PLOS remains concerned about the widespread use of the ethics approval number, particularly since the articles citing this approval report collection of sample types not listed in the approval document.
– PLOS identified potential competing interests between the committee that granted the ethics approval and one or more of the article’s authors.

In light of the unresolved issues, the Expression of Concern stands.

## Supporting Information

S1 File Overview of 55 articles referencing ethics approval number N° 2016–011. Note that the #2016.011 approval document only indicates approval for the use of stool samples although these articles report collection of other samples including urine, oral fluids, sputum, saliva, vaginal swabs, bronchial aspirates, dental plaque, and bronchoalveolar lavage.
(XLSX)
